# The Use of Virtual Reality Technology in the Treatment of Psychopathological Disorders

**DOI:** 10.3390/jcm11185358

**Published:** 2022-09-13

**Authors:** José Gutiérrez-Maldonado

**Affiliations:** Department of Clinical Psychology and Psychobiology, University of Barcelona, Paseo Valle de Hebrón, 171, 08035 Barcelona, Spain; jgutierrezm@ub.edu

Jaron Lanier proposed the name “Virtual Reality” to refer to interactive simulations produced through the use of computer technology, although the idea was formulated in the sixties by a pioneer of computer graphics, Ivan Sutherland [1,2]. In 1968, he laid the foundations that would serve as the basis for the development of the current head mounted displays (HMD). The first experiments with HMDs involved systems that projected the signal received from servo-controlled cameras onto the wearer’s eyes. Using this technique, the company Bell Helicopter developed in the 1960s a headset connected to a night-vision camera that allowed the pilot to land in the dark. In 1966, Ivan Sutherland and Bob Sproull transformed this remote vision system into a virtual reality system, replacing the image provided by the cameras with a virtual environment consisting of a schematic room. Users could ‘enter’ the room, turn their head, and look out in different directions.

Sutherland’s contributions were pioneering in the development of computer graphics; however, restricting the concept of virtual reality to computer technology is not appropriate, since what is important to characterize this technological field is not how a certain objective is achieved, but the objective that is pursued in itself. That objective, which is none other than to produce the illusion of being present in a different place from the one that one really is (currently, we would say that it would also be to produce the illusion of being a different person from the one that one really is) has been achieved in each historical moment with the technological resources available at that time. In the second half of the 19th century, for example, stereoscopic optical technology also pursued this goal.

The delimitation of the factors that define the illusion presence has generated interesting discussions among researchers of virtual reality applications. People who interact with virtual environments do not have the experience of observing them from the outside, but rather that they form part of them. This illusion of presence is similar to other examples of altered perception, such as optical illusions, although it is more complex as it usually includes a large number of stimuli and, in certain systems, a variety of sensory modalities. Although people who experience these illusions know they are not real, they perceive them as if they were. Thus, patients with acrophobia who look down on the street from high above in a virtual environment know that they are not really in that situation, but the sensations experienced and the responses to them are the same as they would be in reality. This is an operational definition of the concept of presence, which in more formal terms can be formulated as the degree to which a person exposed to a virtual environment produces the same behavioural, cognitive, and physiological responses that would be produced in the real situation that is being simulated.

The factors that influence the illusion of presence may be classified according to different criteria. One possible classification distinguishes between variables inherent to the technological system used to create the simulation and personal variables of the user. Another classification considers the relationship between the user and the system and therefore distinguishes between general factors (present in any kind of simulation) and specific factors. In terms of the system used to create the simulation, the diverse range of variables include, for example, the amplitude of the field of view, the frequency with which screen images are presented, and the latency of the system’s response to user actions. Many personal variables also influence the illusion of presence, ranging from the person’s experience of computer technology devices to his cognitive abilities and personality traits [3]. As regards the general factors (present in any sphere of application) that may help to increase the sense of presence in a virtual environment, some of these are perceptual (immersion), while others are motor related (interaction). Those systems that limit the entry of stimuli from the real world and boost those corresponding to the virtual environment reduce, through perceptual mechanisms, the presence of the real world and heighten the presence of the virtual one. Motor variables are also important. If users have the possibility of interacting with the virtual environment (moving around it, touching and/or moving objects, etc.), their illusion of presence will be greater than if they are limited to observing what is happening. In addition to these general factors related to immersion and interaction, each sphere of application will have its own specific factors. In psychopathology, for example, the most important specific factors are what are referred to as clinically significant elements, that is to say, those stimuli and contexts that elicit the problematic response or which modulate its intensity. These elements are highly diverse and are directly related to each specific disorder, and they should form part of a system’s design from the outset as they may have a greater influence than general factors on levels of presence. Knowledge of the psychopathological characteristics of disorders is therefore crucial, as it is this which enables these elements and their properties to be determined.

The balance between internal and external validity is one of the main advantages provided by virtual reality technology. This is the case not only in psychological assessment and treatment but also in research, since it is now possible to present stimuli under conditions characterized by a degree of control equivalent to that obtained when applying traditional procedures in the laboratory or consulting room, at the same time as simulating the natural situations in which these stimuli are present. Consequently, the modification of a certain behaviour can be carried out with high internal validity without undermining external validity, since the learning that takes place in the virtual environment is more easily transferable to real life. For example, techniques of cue exposure intensified via VR allow, at least theoretically, for higher levels of ecological validity, improving the transfer of learning to the natural situation while reducing the logistical complications required for implementation. Furthermore, using this technology, it is also possible to provide patients with new experiences with which to test beliefs about themselves and the environment. In patients with anorexia nervosa, for example, certain beliefs related to food and the body have significant similarities with delusional ideas. Their egosyntonic nature makes them very resistant to any change, which makes the application of the treatment very difficult. Technology can contribute to solving this problem, since there are studies indicating that treatments that incorporate VR technologies result in greater motivation to change and lower drop-out rates [4]. Techniques such as exposure are especially indicated in these patients, as they have a particularly appropriate impact on cognitive rigidity and the difficulty of integrating and generalizing new information. VR technology provides several advantages on this regard, as it allows exposure to be applied in simulations of natural situations, while maintaining the degree of control that is only possible in the context of a laboratory or a hospital. In addition, the recording of different measurements in real time throughout the treatment sessions is facilitated in a particularly effective manner with the use of VR devices, thanks to the degree of control that this technology provides, and this is made possible without detracting from the ecological validity, since these measures are taken while the patient is interacting with the simulation of a natural situation [5]. The manipulation of contexts and their personalization is also made possible, adapting the stimuli and environments to the individual characteristics of the patients. The cues that trigger craving reactions in patients with addictions, for example, are similar to some extent, but there are also individual differences in the substances consumed and in the contexts in which these behaviors occur. Facilitating the adaptation of exposure to these individual patient characteristics should be one of the goals of future VR applications in the treatment of these disorders.

For many years, VR technology was regarded as expensive and complicated, and until very recently, this was indeed the case, at least when it came to immersive systems, although it should be noted that some studies showed that valid and effective outcomes could be achieved in the clinical field with non-immersive and low-cost equipment [6]. Currently, the cost of this technology is no longer an obstacle due to the interest shown by various companies in the consumer electronics sector, who saw the possibilities for growth and, therefore, profits. As a result of their investment in the development and large-scale production of this technology, low-cost and high-quality equipment is now widely available. 

An enormous variety of devices and components have been developed since the birth of computer VR technology, although the most common systems involve variations on the initial idea of an HMD. This kind of device enables screens to be placed very close to the user’s eyes, and with adequate lenses, it is possible to focus vision on them so that the user perceives, stereoscopically, depth in the visual content that is sent from a computer. Another basic component of these systems are sensors that enable information about the user’s head position to be relayed to the computer, thus enabling the selection of the visual information that is sent to the screens in accordance with the user’s orientation. In addition, hearing, touch, and other sensory modalities can be incorporated through the use of headphones, gloves, and other devices. The sensory information that is received depends, therefore, on the user’s actions. This basic concept, referred to as sensorimotor coupling, is central to computer VR technology, and it can be developed and made as complex as necessary depending on the objectives being sought. It is possible, for example, to create a system in which the user can interact with the simulation not merely through changes in head position but also by moving other parts of the body, or even the body as a whole, and thus the simulation becomes significantly more realistic. The information gathered from users does not have to be limited to motor variables; any type of organism response, for example, psychophysiological or verbal, may be used provided that adequate sensors are available. One type of response that is increasingly being used in VR systems nowadays is eye movements, and this enables attentional variables to be measured and cognitive variables to be extrapolated. 

The first studies to examine applications of VR in the field of psychopathology focused mainly on anxiety disorders such as fear of flying [7], acrophobia [8], post-traumatic stress disorder [9], social phobia and fear of public speaking [10,11,12], agoraphobia [7], claustrophobia [13], driving phobia [14], and spider phobia [15]. In those first years, various research reviews [16,17,18,19,20] found evidence of efficacy in the treatment of anxiety disorders. Nevertheless, from the outset, VR technology proved to be useful not only for the purposes of exposure and desensitization in the context of phobias but also as a way of improving cognitive techniques in severe disorders such as anorexia and bulimia [21], as well as for increasing the skills of children with autism spectrum disorders [22]. From those early years to the present, the scope of application of these technologies in psychopathology has grown enormously, including almost any type of disorder: neurodevelopmental disorders, addictions (both substance and behavioral), disorders related to traumas and stressors, sexual dysfunctions, schizophrenia, sleep-wake disorders, depressive disorders, neurocognitive disorders, etc. Studies have been developed on the evaluation of these problems, on their treatment, and also on their causes and mechanisms of manifestation and maintenance.

The 20 papers published in this Special Issue represent a sample of the current state of research in this field, with examples of applications for the study, assessment, and treatment of attention deficit and hyperactivity disorder (ADHD), addictions, depression, anxiety, schizophrenia, anorexia nervosa, bulimia nervosa, body image, virtual body ownership illusions, aggressive behavior, rehabilitation, and pain. An analysis of the ethical issues related to the application of these technologies is also made. 

Regarding ADHD, Areces et al. [23] studied the role that anxiety plays in performance tests. The participants of their research were 103 children between the ages of 6 and 16, who were being explored for the possible presence of ADHD. The results showed that state anxiety influenced attentional performance, which indicates the importance of controlling this influence so that the evaluation is not biased.

Three papers are focused on addictions, in particular alcohol use disorder (AUD). The determinants and predictors of craving constitute one of the main research topics in this field. Hernández-Serrano et al. [24] present the results of a study conducted on 72 outpatients, in which it was observed that the most severe patients experienced higher levels of craving during exposure to virtual environments related to alcohol consumption and to specific alcohol-related cues. It was also found that the patients who perceived the specific alcohol cues more realistically also showed higher levels of craving as a consequence of the exposure. Another study from the same group [25], conducted with 42 outpatients with AUD, concluded that Including VR cue exposure in treatment protocols may provide benefits, mainly among those who show higher levels of craving before treatment. In a narrative review, Lebiecka et al. [26] present recent developments in this topic.

One pilot study from Szczepańska-Gieracha et al. [27] evaluated the application of VR technology in depression. Thirty-five elderly women with depressive symptoms, who had not improved after the application of previous treatments, participated in a trial in which a multimodal treatment program that included VR therapy was applied. The evaluation of the results of the treatment showed that it had managed to reduce the intensity of the depressive symptoms.

One of the best represented disorders in this Special Issue is schizophrenia. Four papers present results related to the application of VR in its study, assessment, and treatment. The recognition of emotions is evaluated by Muros et al. [28]. The difficulties that many patients with schizophrenia show to recognize emotions through facial expression cause problems in their social interaction and in their integration in the community. VR offers interesting possibilities to study these difficulties, and this research, in which 56 patients and 56 controls participated, shows its ability to detect and modify them.

In a proof-of-concept study, Dellazizzo et al. [29] merged cognitive behavioral therapy (CBT) with VR therapy for the treatment of refractory voices, finding a certain synergistic effect. Rus-Calafell et al. [30] present some results from the AVATAR project. This project takes advantage of the possibilities offered by VR to produce a virtual embodiment of auditory hallucinations. In this way, the patient can visualize them and establish a dialogue with them that reduces their negative consequences. The study published in this Special Issue explores the contribution of sense of voice presence to the efficacy of the therapy. Data from 39 patients who received AVATAR therapy and who were followed up for 12 months were analyzed, finding that the interaction between anxiety reduction and voice presence was significant on some of the measures of therapy efficacy.

Another area of application of VR technology in schizophrenia is related to the measurement and improvement of executive functions. The paper from Tyburski et al. [31] performs a critical review of this field, comparing VR tests with traditional tools.

Eating disorders and body image have also been covered by several studies in this Special Issue. The study presented by Porras-García et al. [32] analyzes the efficacy of a VR body exposure therapy added to the usual treatment of anorexia nervosa (AN). Thirty-five patients participated. After the treatment and at follow-up, the experimental group (treatment as usual plus VR exposure) showed significantly better values than the control group (treatment as usual) in the measures of body image disturbances and fear of gaining weight.

The paper by Monthuy-Blanc et al. [33] had two objectives; the first one was to test the discriminant and convergent validity between traditional paper-based figure rating scales (FRS) and the VR-based FRS “eLoriCorps Immersive Body Rating Scale” in the assessment of body image disturbances. The second objective was to explore the contribution of the egocentric VR perspective of eLoriCorps to the understanding of those disturbances. The egocentric perspective differs from the allocentric in that the former is associated with intra-individual comparisons, while the latter is based on inter-individual comparisons.

The study by Porras et al. [34] provides some data on a topic not yet widely studied. Research carried out to date indicates that attentional biases (AB) towards the body are frequent, both in clinical and healthy populations, and that these biases are different in men and women, finding that women show greater biases towards weight related body parts. Few studies have investigated body-related AB in men. This study aimed to assess the presence of muscle-related AB in men and its relationship with muscularity dissatisfaction (MD), using an innovative combination of VR and eye-tracking (ET) technologies. Men with high MD spent more time looking at body parts related to muscularity compared to men with low MD. This study combined VR with ET for the first time in the research of this topic. 

In a narrative review, Matamala-Gómez et al. [35] examine the information available to date on the use of the embodiment phenomenon for clinical purposes and suggest future lines of research in this field.

Previous randomized controlled trials [36,37] have found that innovative interventions based on the application of cue exposure techniques through virtual reality (VR-CET) have been effective for patients in whom the usual treatments have not been sufficient. In order to evaluate the feasibility and acceptability of VR-CET, Nameth et al. [38] recruited adults previously treated for binge eating disorder or bulimia nervosa who still had binge eating behaviors from an outpatient university eating disorder clinic to receive an additional treatment consisting of several sessions of VR-CET. The results obtained showed that the application of VR-CET in real clinical contexts is acceptable and feasible.

The first disorders on which VR application studies appeared were anxiety disorders, in the 1990s, to efficiently perform exposure therapy. In this Special Issue, two articles have focused on these disorders. The purpose of the study by Seok Jeong et al. [39] was to explore whether the administration of CBT through VR-based resources, for the treatment of social anxiety, reached acceptable levels of efficacy with a reduced number of sessions. It was found that treatments lasting 9–10 sessions reached those levels of efficacy.

The meta-analysis by López-Valverde et al. [40] focused on dental anxiety and pain. Positive results were found on the use of VR to reduce these effects of dental treatments in children, even though further research is required since there are still very few studies on this topic.

Rehabilitation has been another of the fields of application of VR technologies in which work has been carried out since the early years. In the paper by Józwik et al. [41], the aim was to assess the efficacy of VR-enhanced cardiac rehabilitation (CR) to reduce the symptoms of anxiety and depression suffered by a large number of patients undergoing ambulatory phase II of CR. The data from 77 participants showed positive results. Chronic patients frequently experience symptoms of depression and anxiety, due to the lack of control they have over their pathologies. Depression has been estimated to occur in 27–79% and anxiety in 21–96% of patients with chronic obstructive pulmonary disorder (COPD). Rutkowski et al. [42] compared two groups that received a traditional pulmonary rehabilitation (PR) programme. Additionally, one group received 10 sessions of Schultz autogenic training, and the other received 10 sessions of immersive VR-therapy. The results showed a reduction in stress levels only in the VR-group, and the symptoms of anxiety and depression were significantly reduced only in the VR-group, with a strong effect size. 

New applications of VR in psychopathology appear continuously. One of them is aimed at treating antisocial behavior, aggressiveness, and violence. Klein Tuente et al. [43] investigated the effectiveness of a VR-based aggression prevention training. In this randomized controlled trial at four Dutch forensic psychiatric centers, 128 inpatients participated. The results suggest that this training does not decrease aggressive behavior in forensic inpatients; however, there are indications that this intervention temporarily influences hostility, impulsivity, and anger control skills.

The increasing use of VR in clinical practice opens up a series of new concerns related to ethical issues. Thomas D. Parsons [44] considered a number of these concerns. His considerations can guide the use of these technologies in clinical research and practice. Legal and ethical issues, maintaining patient privacy, and the appropriate use of technologies are discussed. In addition, ethical issues must be considered by clinicians when using technologies and algorithms that can extend the patients cognitive abilities.

This Special Issue includes very diverse studies: randomized controlled trials, nonrandomized trials, pilot trials, proofs of concept, systematic reviews, meta-analyses, narrative reviews, and articles with theoretical and ethical considerations. As a whole, they show the diversity of topics and methods currently used to investigate a field in constant growth, such as the applications of virtual reality in psychopathology, and the wide variety of applications for evaluation and treatment. Many of them allow more efficient administration of traditional techniques, whose efficacy and effectiveness have already been firmly established. Others make it possible to resolve the dilemma between maximizing internal validity or external validity, facilitating methods that allow a high degree of control of variables to be achieved while at the same time increasing the extrapolation of results to natural contexts. On the other hand, embodiment techniques allow going beyond the illusion of presence related to contexts and environmental stimuli, producing not only the illusion of being in a certain environment but also the illusion of being different individuals. The consequences that these applications of VR technology will have for research and for applied practice in psychopathology will open up new opportunities that will hopefully improve our understanding of psychopathological disorders and the possibilities of intervention.
